# Hydrogen-fueled CO_2_ reduction using oxygen-tolerant oxidoreductases

**DOI:** 10.3389/fbioe.2022.1078164

**Published:** 2023-01-05

**Authors:** Jaehyun Cha, Hyeonseon Bak, Inchan Kwon

**Affiliations:** ^1^ School of Materials Science and Engineering, Gwangju Institute of Science and Technology (GIST), Gwangju, South Korea; ^2^ Research Center for Innovative Energy and Carbon Optimized Synthesis for Chemicals (Inn-ECOSysChem), Gwangju Institute of Science and Technology (GIST), Gwangju, South Korea

**Keywords:** hydrogen, carbon dioxide, formate, oxygen-tolerant, hydrogenase, formate dehydrogenase

## Abstract

Hydrogen gas obtained from cheap or sustainable sources has been investigated as an alternative to fossil fuels. By using hydrogenase (H_2_ase) and formate dehydrogenase (FDH), H_2_ and CO_2_ gases can be converted to formate, which can be conveniently stored and transported. However, developing an enzymatic process that converts H_2_ and CO_2_ obtained from cheap sources into formate is challenging because even a very small amount of O_2_ included in the cheap sources damages most H_2_ases and FDHs. In order to overcome this limitation, we investigated a pair of oxygen-tolerant H_2_ase and FDH. We achieved the cascade reaction between H_2_ase from *Ralstonia eutropha* H16 (ReSH) and FDH from *Rhodobacter capsulatus* (RcFDH) to convert H_2_ and CO_2_ to formate using *in situ* regeneration of NAD^+^/NADH in the presence of O_2_.

## 1 Introduction

The development of renewable energy technologies to replace fossil fuels is essential for the sustainable growth of the economy and society. Hydrogen (H_2_), obtained from various sources such as solar (Song et al., 2022), algae (Wang and Yin, 2018), biomass (Lepage et al., 2021), and by-product gas (Lee and Elgowainy, 2018), is expected to be an alternative fuel with high gravimetric energy density and net-zero carbon dioxide (CO_2_) production (Eppinger and Huang, 2017). However, owing to the low volumetric energy capacity of H_2_, its transportation and storage as a fuel are limited (Eppinger and Huang, 2017). Therefore, converting H_2_ into a chemical with a high volumetric energy capacity while maintaining the molar energy capacity is advantageous for the commercialization of alternative energy.

Suitable materials for converting H_2_ energy should satisfy the following conditions: 1) high energy/volume capacity, 2) low energy loss during the conversion process, 3) liquid material at ambient pressure and temperature, and 4) non-flammable chemicals for safety. Thus, the conversion of H_2_ and CO_2_ into formate is an appropriate approach (Ping et al., 2013; Eppinger and Huang, 2017; Mihet et al., 2020). Because formate is a non-flammable liquid at ambient temperature and pressure, it is convenient to transport and store. However, the reaction requires a catalyst, and synthetic catalysts cannot be applied to various H_2_ sources because of their low selectivity, low efficiency, and requirement for precious metals (Loges et al., 2008; Kuehnel et al., 2015; Sordakis et al., 2018). Thus, the substrate specificity and high reaction rate of the enzymatic process reveal the potential for H_2_ and CO_2_ as alternative synthetic catalysts. The oxidation of H_2_ and reduction of CO_2_ occur in hydrogenase (H_2_ase) (Lubitz et al., 2014) and formate dehydrogenases (FDH) (Appel et al., 2013; Amao, 2018; Moon et al., 2020), respectively, among oxidoreductases.

Formate hydrogenlyase (FHL) is a natural FDH and H_2_ase-linked enzyme complex that catalyzes formate/H_2_ interconversion (McDowall et al., 2014). The catalytic bias of the FHL is H_2_ production from the oxidation of formate (Pinske and Sargent, 2016; Schwarz et al., 2018). The semi-artificial coupling of H_2_ase and FDH from *Desulfovibrio vulgaris* Hildenborough successfully demonstrated the interconversion of H_2_ and CO_2_ into formate (Sokol et al., 2019). Cheap and sustainable H_2_ sources, such as coke oven gas generated from steel industries, contain a small portion of O_2_ (0.4–1.7%) (Li et al., 2019; García García et al., 2020). Because of the transition metal active sites and low potential electrons, most H_2_ases and FDHs are inhibited or irreversibly damaged by a trace amount of O_2_ (Fontecilla-Camps et al., 2007; Niks and Hille, 2018), limiting the application of H_2_ conversion obtained from various renewable sources.

**FIGURE 1:**
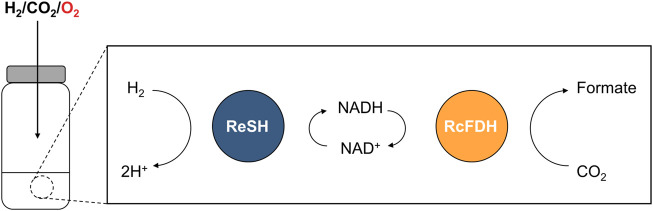
Schematic showing the NAD^+^-dependent cascade reaction of ReSH and RcFDH in the presence of O_2_.

H_2_ases and FDHs from aerobic organisms maintain their catalytic activities under aerobic conditions. H_2_ase can be classified according to the metal ion composition of their active sites in [NiFe], [FeFe], and [Fe] H_2_ases (Lubitz et al., 2014). [NiFe] H_2_ase inactivation under aerobic condition was shown to form an inactive state by O_2_ bridging to Ni-Fe through X-Ray crystallography, electron paramagnetic resonance (EPR) studies, and the density function theory calculations (Volbeda et al., 2005; Shafaat et al., 2013; Qiu et al., 2018). Well-studied O_2_-tolerant [NiFe] H_2_ases from *Escherichia coli* (Sargent, 2016), *Aquifex aeolicus* (Pandelia et al., 2010), and *Ralstonia eutropha* (Burgdorf et al., 2005) have potential biotechnological applications such as biofuel cells and H_2_ production. Their O_2_ tolerance was attributed to the reduction of O_2_ bound to NiFe active site into either hydrogen peroxide or water (Lauterbach and Lenz, 2013; Wulff et al., 2014; Horch et al., 2015). The soluble H_2_ase from the *R. eutropha* (ReSH) complex contains heterodimeric [NiFe] hydrogenase (HoxHY) subunits and diaphorase (HoxFU) subunits, which reduce NAD(P)^+^ while oxidizing H_2_ (Lauterbach and Lenz, 2013). FDH can be classified according to the metal ion composition of their active sites in molybdenum (Mo) containing and tungsten (W) containing formate dehydrogenase (Moon et al., 2020). Under aerobic conditions, the inactivation of FDH occurs by substitution of oxo ligand for sulfide ligand at the active site by O_2_ (Duffus et al., 2020). It was proposed that the O_2_ tolerance of W-containing FDH2 from *Desulfovibrio vulgaris* Hildenborough results from reduction of O_2_ to hydrogen peroxide by formate oxidase activity (Graham et al., 2022). The FDHs from *Clostridium carboxidivorans* strain P7T (Alissandratos et al., 2013), *Methylobacterium extorquens* AM1 (Laukel et al., 2003; Baccour et al., 2020), and *Rhodobacter capsulatus* (Hartmann and Leimkühler, 2013) maintain high CO_2_-reducing activity under aerobic conditions. FDH from *R. capsulatus* (RcFDH) consists of FdsA subunit containing the bis(molybdopterin guanine dinucleotide) cofactor and FdsGB diaphorase subunit for oxidizing NADH while reducing CO_2_. We hypothesized that H_2_ and CO_2_ are converted to formate through a cascade reaction of O_2_-tolerant H_2_ases and FDHs under oxic conditions. Here, we demonstrated a cascade reaction of ReSH and RcFDH with NAD^+^ regeneration (Figure 1). Formate production was observed under anaerobic and O_2_ concentration-controlled conditions.

## 2 Materials and methods

### 2.1 Materials

The 5X In-Fusion^®^ HD Enzyme Premix was purchased from Takara Bio (Kusatsu, Japan). Strep-Tactin XT 4 Flow high-capacity resin was obtained from IBA Life Sciences (Göttingen, Germany). Disposable PD-10 desalting columns were purchased from Cytiva (Marlborough, MA, United States). Vivaspin 6 centrifugal concentrators with a molecular weight cutoff (MWCO) of 100 kDa were purchased from Sartorius (Göttingen, Germany). A polypropylene column (1 ml) was purchased from Qiagen (Hilden, Germany). The Ziptip C_18_ resin was purchased from Millipore (Burlington, MA, United States). All other chemical reagents were purchased from Sigma-Aldrich (St. Louis, MO, United States) unless otherwise stated.

### 2.2 Construction of plasmids and strains

To construct the strep-tag II-fused RcFDH expression plasmid, pTrcHis-RcFDH (Choi et al., 2018) was used as a template. Infusion cloning was performed to substitute the hexahistidine-tag for strep tag II. pTrcHis-RcFDH was amplified by PCR with the in-fusion primer (FW: 5′-GCC​ACC​CGC​AGT​TCG​AAA​AAG​GTA​TGG​CTA​GCA​TGA​CGG​ATA​CC-3′, RV: 5′-CGA​ACT​GCG​GGT​GGC​TCC​AAG​AAC​CCC​CCA​TGG​TTT​ATT​CCT​CC-3′). The PCR product was mixed with 5X In-Fusion HD Enzyme Premix to generate pTrcHis-strep-RcFDH. The *E. coli* MC1061 strain was transformed with pTrcHis-Strep-RcFDH, and the *R. eutropha* HF210 [pGE771] strain (Lauterbach and Lenz, 2013) was used as the ReSH-expressing strain.

### 2.3 Expression of ReSH and RcFDH

For the expression of ReSH and RcFDH, a 7 L scale fermenter was used. Previously, Lenz described the heterotrophic cultivation of *R. eutropha* derivatives (Lenz et al., 2018). A 10X H16 buffer (pH 7.0) consisting of 250 mM Na_2_HPO_4_ and 110 mM KH_2_PO_4_ was used as the medium. For a 1 L of fructose-ammonium (FN) medium, 100 ml of 10X H16 buffer was mixed with 850 ml of sterilized water (additional 13% (w/v) of Bacto agar in case of solid agar plates) and autoclaved. Next, 10 ml of 20% (w/v) NH_4_Cl, 1 ml each of 20% (w/v) NH_4_Cl, 20% (w/v) MgSO_4_*7H_2_O, 1% (w/v) CaCl_2_*H_2_O, 0.5% (w/v) FeCl_3_*6H_2_O (in 0.1 N HCl), 1 mM NiCl_2_, and 1.25 ml of 40% (w/v) D-fructose were mixed and filled up to 1000 ml with sterile H_2_O. A single colony of *R. eutropha* was pre-cultured in 50 ml of FN medium containing 10 μg ml^−1^ tetracyclin until the OD436nm reached 1. For the main culture, 5 L of modified fructose-glycerol-ammonium (FGN_mod_) with 0.05% (w/v) glycerol, 5 ml of SL6 trace element solution (Lenz et al., 2018), and 5 ml of 1 mM ZnCl_2_ (added to the FN medium containing 10 μg/ml tetracycline) were prepared in the fermenter. The pre-culture was inoculated into the FGN_mod_ medium and subjected to 300 rpm shaking and 1 VVM aeration at 30°C. The pH range was maintained between 6.9 to 7.0 through automatic injection of 1 N NaOH. After 24 h, 5 ml of 1 mM NiCl_2_ was added. When the OD at 436 nm reached 9–11, the cells were harvested by centrifugation at 6,000 × g for 10 min before storage at −80°C.

For RcFDH expression, a single-cell colony was pre-cultured in Luria-Bertani (LB) medium containing 150 μg ml^−1^ ampicillin for 12 h at 37°C. For the main culture, 5 L of LB medium containing 150 μg ml^−1^ ampicillin, 1 mM sodium molybdate, and 20 μM isopropyl β-D-1-thiogalactopyranoside was prepared in the fermenter. The pre-culture was inoculated into the LB medium and subjected to 100 rpm shaking and 0.1 VVM aeration at 30°C. After 24 h, the cells were harvested by centrifugation at 6,000 × g for 10 min before storage at -80°C.

### 2.4 Purification of ReSH and RcFDH

**FIGURE 2:**
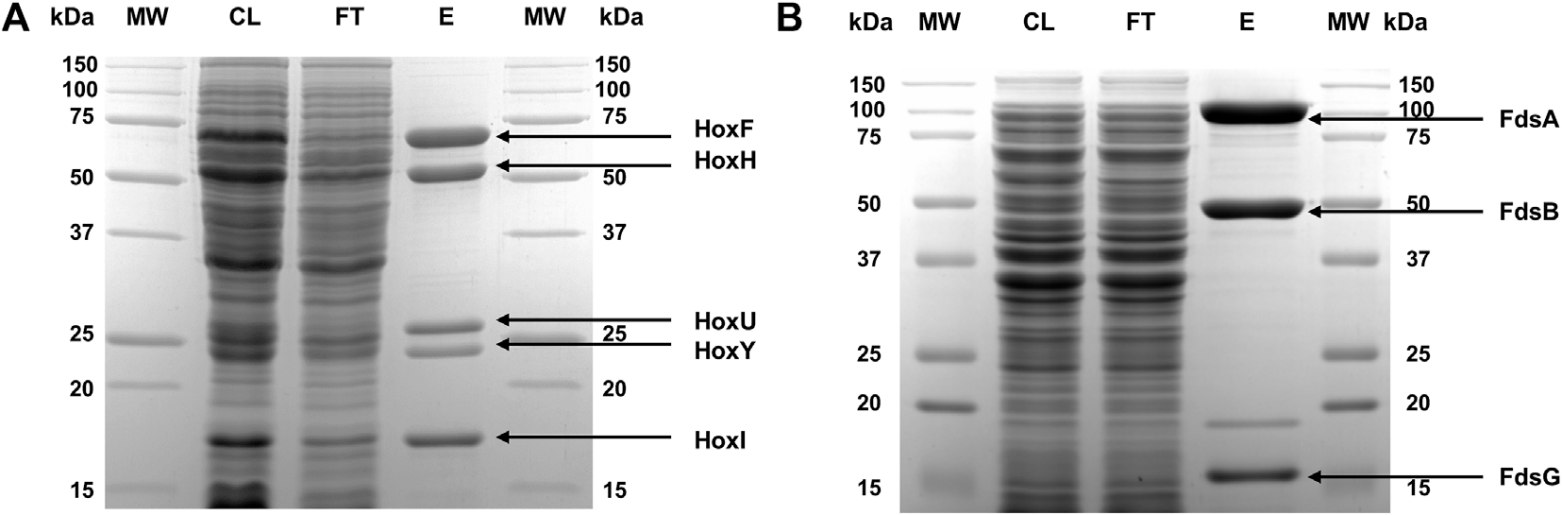
SDS-PAGE of purified proteins. **(A)** ReSH and **(B)** RcFDH stained with Coomassie blue. The lanes are molecular weight markers (MW), cell lysate after sonication (CL), flow-through streptavidin resin (FT), and eluted protein (E).

To purify ReSH and RcFDH, cell pellets were resuspended in 50 mM potassium phosphate buffer (pH 7.0) (Kpi buffer) containing 1 mg/ml lysozyme to a concentration of 1 g/10 ml. The resuspended cells were lysed by sonication (amplitude 28%, on/off 2 s/4 s) for 1 h. Insoluble cell debris was removed by centrifugation at 13,000 × g for 30 min. Strep-Tactin XT 4Flow high-capacity resin (2 ml) was mixed with the clear supernatants and incubated at 4°C for 30 min. The resin was washed with Kpi buffer containing 300 mM potassium chloride on a gravity-flow polypropylene column to remove any impurities. The proteins were eluted with 3 ml of Kpi buffer containing 50 mM biotin and buffer-exchanged with Kpi buffer containing 10 mM potassium nitrate using a PD-10 column. Protein purity was verified by SDS-PAGE (Figure 2). The concentrations of purified proteins were determined by measuring their absorbance at 280 nm using a microplate reader (Synergy, BioTek, Winooski, VT, United States), as previously reported for other proteins (Kim et al., 2019; 2021; Bak et al., 2020). The extinction coefficients of ReSH and RcFDH were calculated to be 165,710 and 350,000 M^−1^⋅cm^−1^, respectively, based on their amino acid sequences.

### 2.5 Matrix-assisted laser desorption ionization–time of flight (MALDI-TOF) mass spectrometry

Proteins in buffer were desalted using Ziptip C_18_ according to the manufacturer’s protocol. Purified ReSH and RcFDH were mixed in a 1:1 (v/v) ratio with a sinapinic acid-saturated matrix solution consisting of 30% acetonitrile, 0.1% trifluoroacetic acid (TFA), and 70% water (v/v). The mixtures were subjected to mass characterization by Autoflex speed (Bruker Corporation, Billerica, United States).

### 2.6 Enzyme kinetics

The enzyme reaction kinetics of ReSH were measured for the NAD^+^-dependent oxidation of H_2_ to H^+^ in the presence or absence of O_2_. The sealing cuvette was filled with 900 μL of Kpi buffer containing NAD^+^ and sealed; then, 100% H_2_ and a mixed gas consisting of 10% O_2_ and 90% N_2_ (or 100% N_2_ for anaerobic conditions) were injected simultaneously for 30 min at 10 ml/min. ReSH (2 ml, 80 nM) was purged with 10 ml/min N_2_ gas bubbling in a 10 ml sealing vial for 30 min to remove O_2_ from the air. The reaction was initiated by mixing 100 μL of 80 nM ReSH with a gas-saturated solution in a sealed cuvette. The final concentration of NAD^+^ was varied from 0 to 2 mM.

The enzyme reaction kinetics of RcFDH were measured for NADH-dependent reduction of CO_2_ to formate in the presence or absence of O_2_. The sealing cuvette was filled with 900 μL of Kpi buffer containing NADH and sealed; then, 100% CO_2_ and a mixed gas consisting of 4% O_2_ and 96% N_2_ (or 100% N_2_ for anaerobic conditions) were injected simultaneously for 30 min at 10 ml/min, respectively. RcFDH (2 ml, 2 μM) was purged with 10 ml/min N_2_ gas bubbling in a 10 ml sealing vial for 30 min to remove O_2_ from the air. The reaction was initiated by mixing 100 μL of 2 μM RcFDH with a gas-saturated solution in a sealing cuvette. The final concentration of NADH was varied from 0 to 1 mM.

All measurements were performed in triplicate based on the change in the absorbance at 365 nm in the cuvette, measured using a T60 UV-Vis spectrophotometer (PG Instruments Ltd., Lutterworth, UK). The change in absorbance over 1 min was plotted using the Michaelis-Menten equation to calculate the kinetic parameters.

### 2.7 Formate production and quantification

For the cascade reaction in the presence or absence of O_2_, the gas content was controlled in a 20 ml polytetrafluoroethylene (PTFE) septa sealing vial. The vials were filled with 500 μL of reaction solution containing 3.2 U/mL ReSH, 0.16 U/mL RcFDH, 1 mM NAD^+^, and 0.5 M Kpi buffer and sealed. A needle was inserted into the septa for gas evacuation. Then, 10 ml/min CO_2_ and 20 ml/min N_2_/O_2_ mixed gas were injected for 30 min (the needle did not enter the reaction solution). The O_2_ ratios of the mixed gas varied from 0%–2%–4%; therefore, the final concentrations of O_2_ were 0, 1, and 2%. The reaction was initiated by a 10 ml/min H_2_ gas injection. Formate production was sampled every 20 min during incubation for 1 h, and 10 μL of 6 N H_2_SO_4_ was added to the 100 μL sample to inactivate the enzymes immediately. Additionally, 240 μL of distilled water was mixed with the sample, and the aggregate enzymes were removed by centrifugation at 13,000 × g. Formate production was quantified by HPLC (1260, Agilent, CA, United States) equipped with a diode-array detector and an Aminex HPX-87H column (BIO-RAD, CA, United States) with a mobile phase of 5 μM H_2_SO_4_ at a flow rate of 0.6 ml/min. The retention time of formate was 13.010 min. The formate concentration was calculated using a formate calibration curve (Supplementary Figure S1).

## 3 Results and discussion

### 3.1 Preparation of ReSH and RcFDH

ReSH and RcFDH are expressed in *R. eutropha* and *E. coli*, respectively. They were purified using affinity resins, as described in the Materials and methods. Five bands of purified ReSH subunits were observed, which matched the expected molecular weights (HoxF, 68,110 Da; HoxH, 54,863 Da; HoxU, 26,173 Da; HoxY, 22,881 Da; HoxI, 18,567 Da) (Figure 2A). Similarly, three bands of purified RcFDH subunits were observed, which were consistent with the expected molecular weights (FdsA, 104,466 Da; FdsB, 52,699 Da; FdsG, 17,304 Da) (Figure 2B). Both enzymes showed high purity. The identity of the purified enzymes was confirmed by MALDI-TOF mass spectrometry. The experimentally determined masses of ReSH subunits were 67,542, 54,492, 26,038, 22,836, and 18,545 m/z, which matched well with the expected masses (68,111, 54,864, 26,174, 22,882, and 18,568 m/z, respectively) with less than 1% deviation (Supplementary Figures S2A–C). The experimentally determined masses of RcFDH subunits were 104,259, 52,385, and 17,136 m/z, which matched well with the expected masses (104,467, 52,700, and 17,305 m/z, respectively) with less than 1% deviation (Supplementary Figures S2D, E). These results showed that the purified ReSH and RcFDH were successfully prepared.

### 3.2 Enzyme kinetics in the presence or absence of O_2_

**FIGURE 3:**
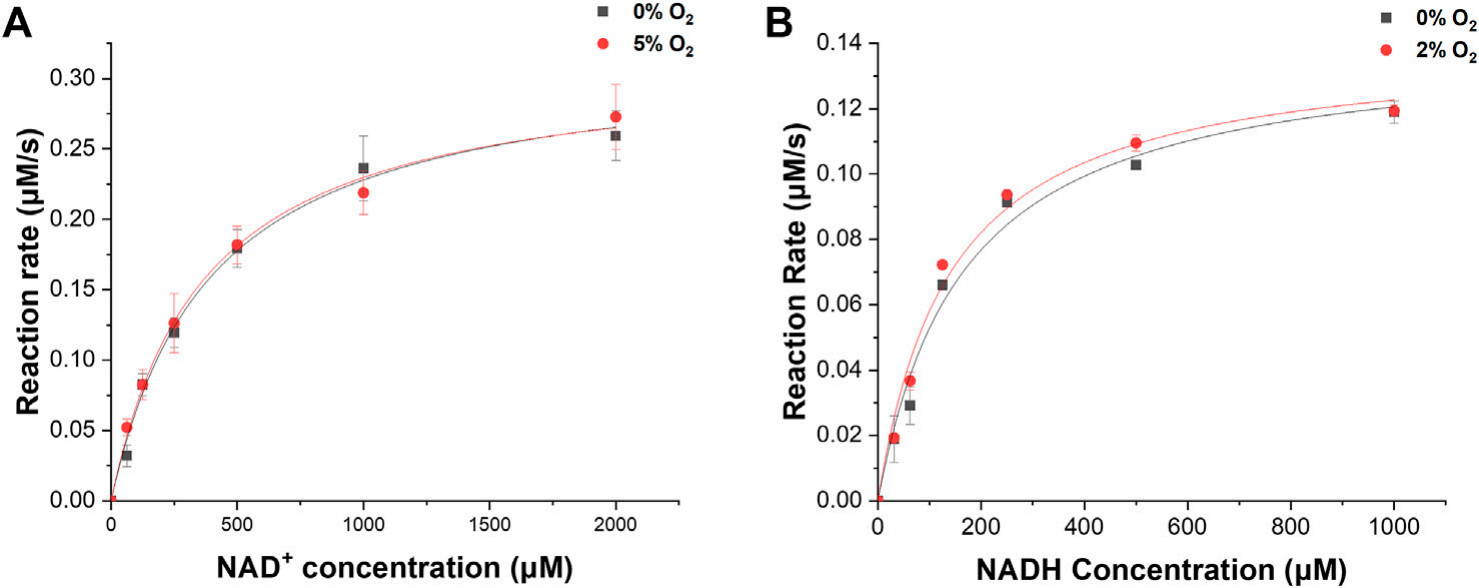
Kinetic analysis. **(A)** NAD^+^-dependent H_2_ oxidation of ReSH in the presence of O_2_, or not. **(B)** NADH-dependent CO_2_ reduction of RcFDH in the presence of O_2_, or not.

We investigated the enzymatic activities of ReSH and RcFDH in the presence or absence of O_2_. The NAD^+^-dependent H_2_ oxidation reaction rate by ReSH was measured, and the Michaelis-Menten curve was fitted to calculate the kinetic parameters using Origin 2022 program (Figure 3A). Both *k_cat_* and *K_m_* values of ReSH showed an insignificant difference under the 0% and 5% O_2_ conditions (Table 1). Similarly, The NADH-dependent CO_2_ reduction reaction rate by RcFDH was measured, and the Michaelis-Menten curve was fitted to calculate the kinetic parameters (Figure 3B). Likewise, *k_cat_* and *K_m_* values of RcFDH showed an insignificant difference between the 0% and 2% O_2_ conditions (Table 2). These results show that purified ReSH and RcFDH retained the enzymatic activity at least under less than 2% O_2_.

**TABLE 1:** Kinetic parameters of ReSH under presence of O_2_ or not.

O_2_ concentration (%)	*k*_*cat*_ (s^−1^)	*K*_*m*_ (mM) (NAD^+^)
0	39.7 ± 1.5	0.393 ± 0.041
5	39.2 ± 1.3	0.364 ± 0.033

**TABLE 2:** Kinetic parameters of RcFDH under presence of O_2_ or not.

O_2_ concentration (%)	*k*_*cat*_ (s^−1^)	*K*_*m*_ (mM) (NADH)
0	0.703 ± 0.043	0.166 ± 0.030
2	0.699 ± 0.035	0.141 ± 0.022

### 3.3 Cascade reaction condition control

We determined the NAD^+^, ReSH, and RcFDH contents for the cascade reaction of ReSH and RcFDH. Owing to the relatively low *k_cat_* value (Tables 1, 2), the rate-determining step was the CO_2_ reduction by RcFDH. Because the reaction rate of RcFDH was saturated at NADH concentrations above 1 mM (Figure 3B), the NAD^+^ concentration was determined to be 1 mM. For the continuous CO_2_ reduction by RcFDH, the concentration of ReSH was determined to maintain a state in which all NAD^+^ was reduced to NADH. The concentration of RcFDH was fixed at 0.08 U/mL and the amount of ReSH was adjusted to 0, 0.08, 0.8, and 1.6 U/mL (U/mL ratio of ReSH:RcFDH = 0:1, 1:1, 5:1, 10:1, 20:1). Reaction solutions were placed in a 20 ml sealing vial, and 10 ml/min CO_2_ and 10 ml/min H_2_ were injected for 1 h simultaneously, after which formate was measured (Supplementary Figure S3). Formate production was not observed in the reaction solution without ReSH. In contrast, substantial formate production was observed in the reaction solution with the three components (ReSH, RcFDH, and NAD^+^). Formate production was saturated above a 5:1 ratio. At higher ReSH concentrations, NAD^+^ was immediately converted to NADH through H_2_ oxidation. This result set the cascade reaction content to 1 mM NAD^+^, and the U/mL ratio of ReSH:RcFDH = 20:1.

### 3.4 Formate production under O_2_ conditions

**FIGURE 4:**
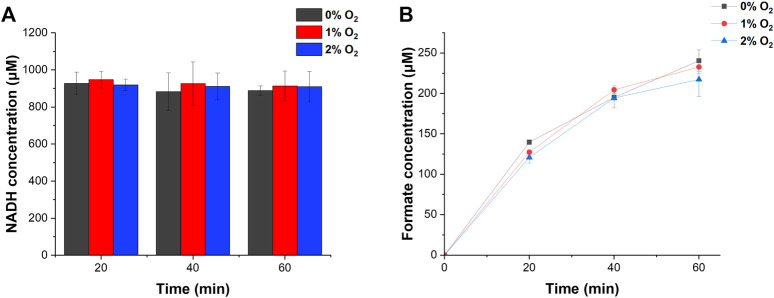
H_2_ and CO_2_ conversion into formate by NAD^+^-dependent ReSH and RcFDH cascade reactions. **(A)** NADH concentration. **(B)** Formate concentration. All measurements were performed in triplicate.

We demonstrated H_2_ and CO_2_ conversion into formate under 0%–2% O_2_ conditions. ReSH, RcFDH, and 1 mM NAD^+^ were mixed and placed in a 20 ml sealing vial. Changes in the concentrations of NADH and formate over time were investigated when O_2_ (at a controlled concentration), H_2_, and CO_2_ were simultaneously and continuously injected into the vial. During the injection of the gases, under all O_2_ conditions from 0% to 2%, NAD^+^ was reduced to NADH and maintained at 1 mM by H_2_ oxidation of ReSH (Figure 4A). Furthermore, the formate concentration increased continuously (Figure 4B) owing to the CO_2_ reduction of RcFDH. Approximately 230 μM of formate was produced after 1 h, which showed a statistically insignificant difference at 0, 1, or 2% O_2_ conditions (*p* > 0.05). In order to investigate the O_2_-tolerant limit of the system, we tested the formate production in a higher concentration of O_2_ (Supplementary Figure S4). We observed a substantial reduction in formate production at 5% O_2_ compared to 0%. Therefore, in the specific enzyme systems we chose, the O_2_-tolerance limit was between 2% and 5%. The O_2_-tolerance of both H_2_ase and FDH is attributed to the reduction of O_2_ bound to the active site of enzymes, leading to the reactivation of active site. Therefore, we speculated that the substantial loss of enzymatic activities at 5% O_2_ results from that O_2_ binding to the active site is more favorable than O_2_ reduction at the active site. These results demonstrate, as hypothesized, the plausibility of a cascade reaction using ReSH and RcFDH, even in the presence of O_2._ Of course, greater O_2_-tolerance limit would be beneficial in developing practical processes. We speculate that there are ways to increase the O_2_-tolerance limit of enzymes. First, the enzyme concentration can be adjusted to increase O_2_-tolerance limit. O_2_-tolerance is likely attributed to the reduction mechanism of O_2_ to either H_2_O or H_2_O_2_. In this case, O_2_ is a co-substrate of these enzymes. Therefore, if the concentrations of enzymes were sufficiently high, the enzymes would quickly reduce O_2_, leading to the increased O_2_-tolerance limit. Another possible approach to increase O_2_-tolerance is engineering enzyme. Recently it was reported that the simple point mutations in the gas tunnel region of O_2_-sensitive CO dehydrogenase greatly increased the O_2_-tolerance limit (Kim et al., 2022). We speculate that such enzyme engineering strategy can be applied to ReSH and RcFDH to increase O_2_-tolerance limit.

## 4 Conclusion

We demonstrated the conversion of H_2_ and CO_2_ into formate using an NAD^+^-dependent cascade reaction of O_2_-tolerant H_2_ase and FDH in the presence of O_2_. However, in order to produce formate using H_2_ and CO_2_ obtained from cheap sources, such as by-product gas from steel industries, we may need to tackle other obstacles. For instances, it was reported that H_2_ases are often damaged by CO (Bagley et al., 1994; Vincent et al., 2007), one of components in by-product gas. We plan to investigate the enzymatic process which is tolerant to both O_2_ and CO in future. Furthermore, we could not obtain the kinetic parameters for both CO_2_ and H_2_ due to difficulty in determining the actual concentration of the gases in the aqueous reaction solution. We plan to determine the kinetic parameters for CO_2_ and H_2_ once suitable gas control facilities are in place.

## Data Availability

The original contributions presented in the study are included in the article/Supplementary Material, further inquiries can be directed to the corresponding author.
