# Supplementary material for: Hydrogen-fueled CO2 reduction using oxygen-tolerant oxidoreductases
Source: Front Bioeng Biotechnol. 2023 Jan 5;10:1078164. doi: 10.3389/fbioe.2022.1078164 (PMC9849572; doi:10.3389/fbioe.2022.1078164)
Supplement: Supplementary file 1 [file DataSheet1.PDF]

Supplementary Material

1 Supplementary Figures

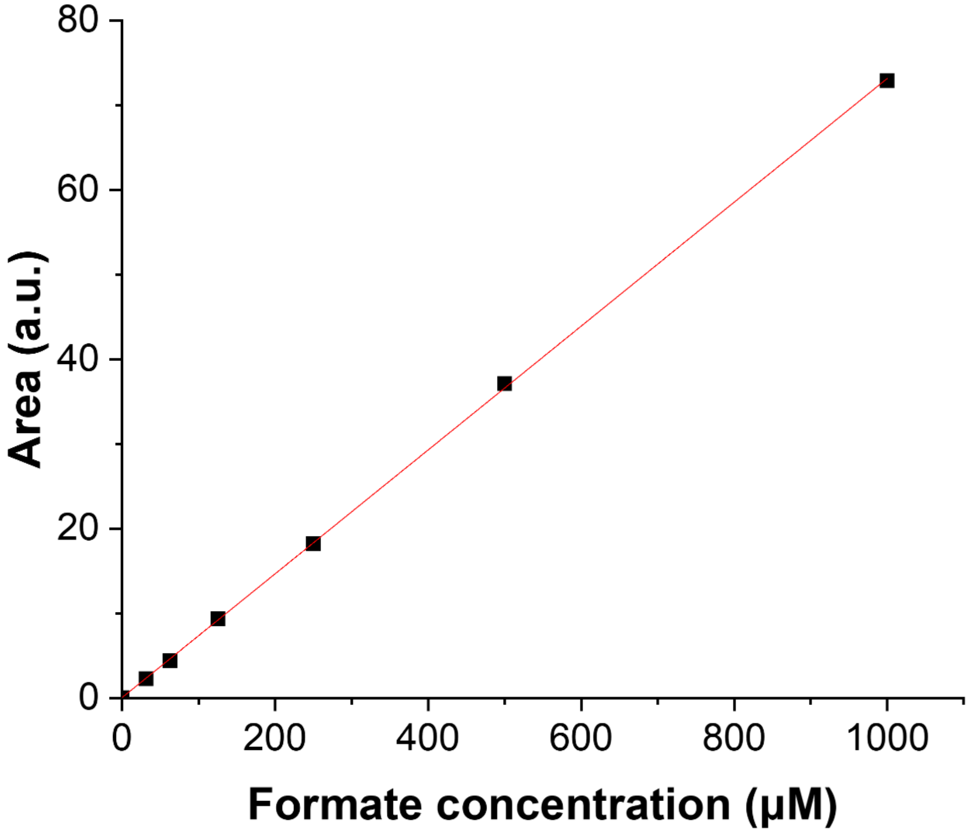

Supplementary Figure 1. HPLC calibration curve of formate ranging from 0 to 1000 µM.

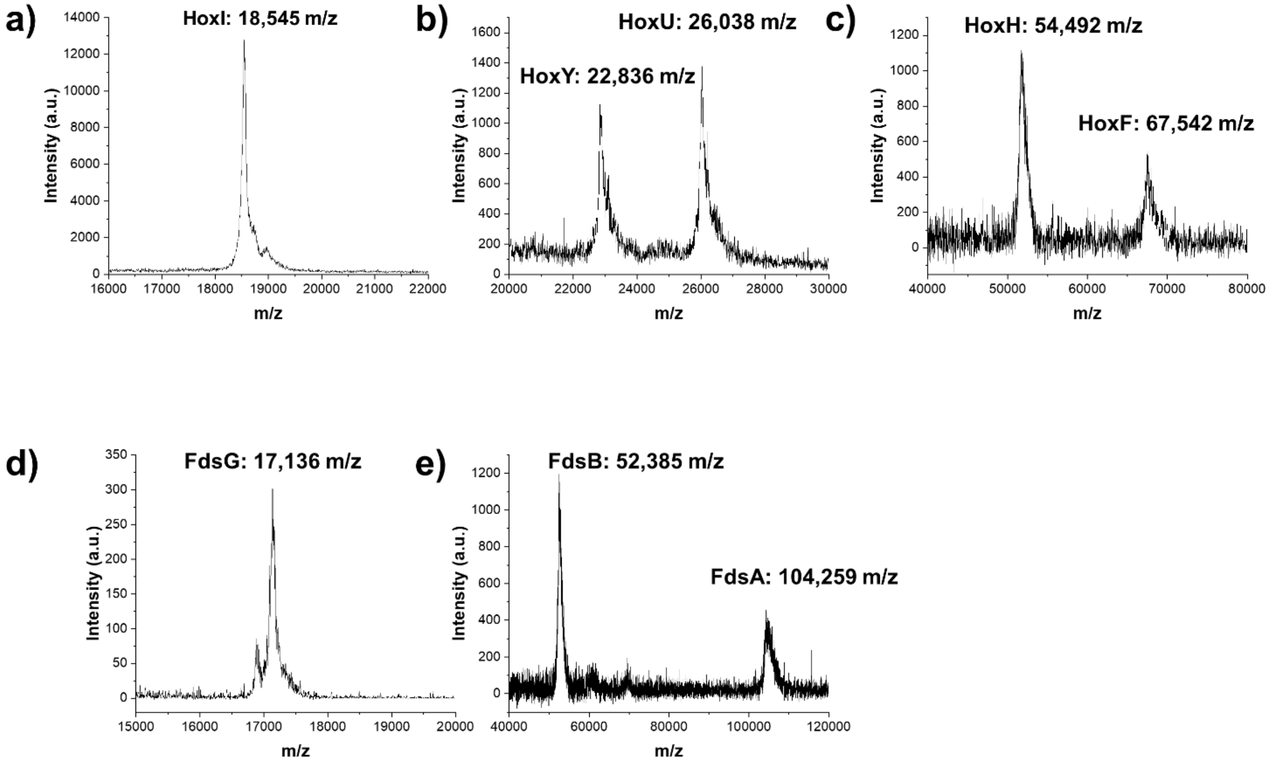

Supplementary Figure 2. MALDI-TOF MS spectra. a) HoxI, b) HoxY and HoxU, and c) HoxH and HoxF subunits of ReSH. d) FdsG and e) Fds A and FdsB subunits of RcFDH.

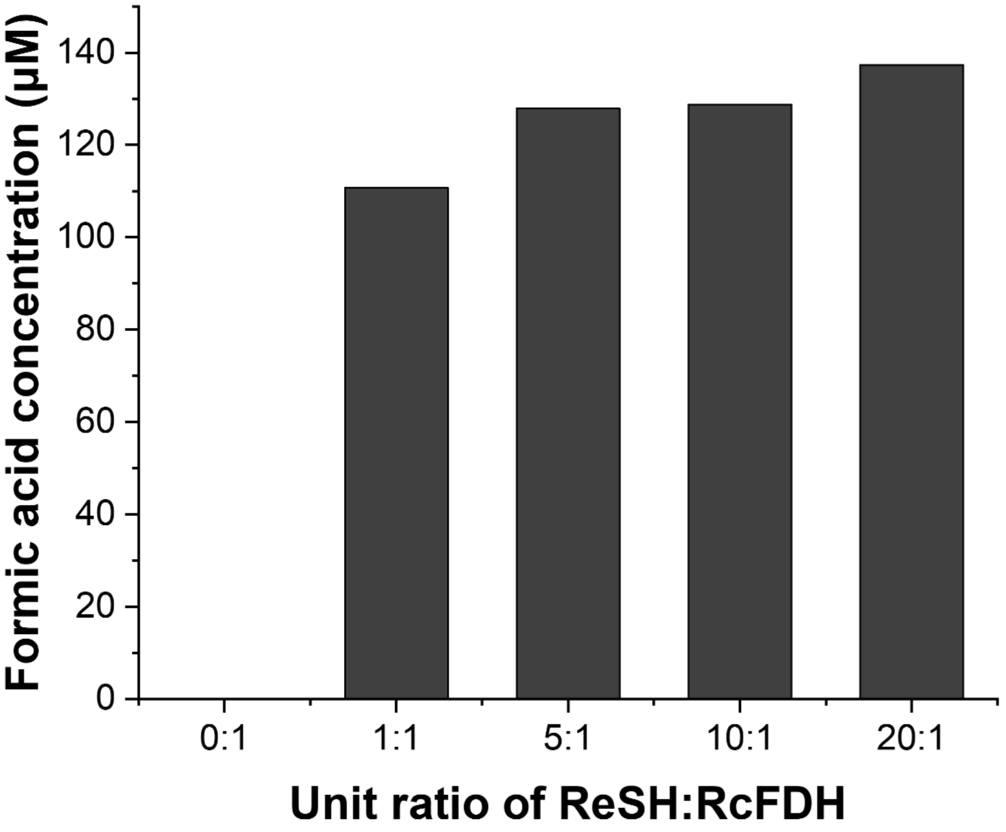

Supplementary Figure 3. Formate produced during 1 h at different ratios of ReSH to RcFDH. ReSH was varied with 0, 0.08, 0.04, 0.8, and 1.6 U/mL (ReSH:RcFDH = 0:1, 1:1, 5:1, 10:1, 20:1) in the presence of 0.08 U/mL RcFDH and 1 mM NAD^+.

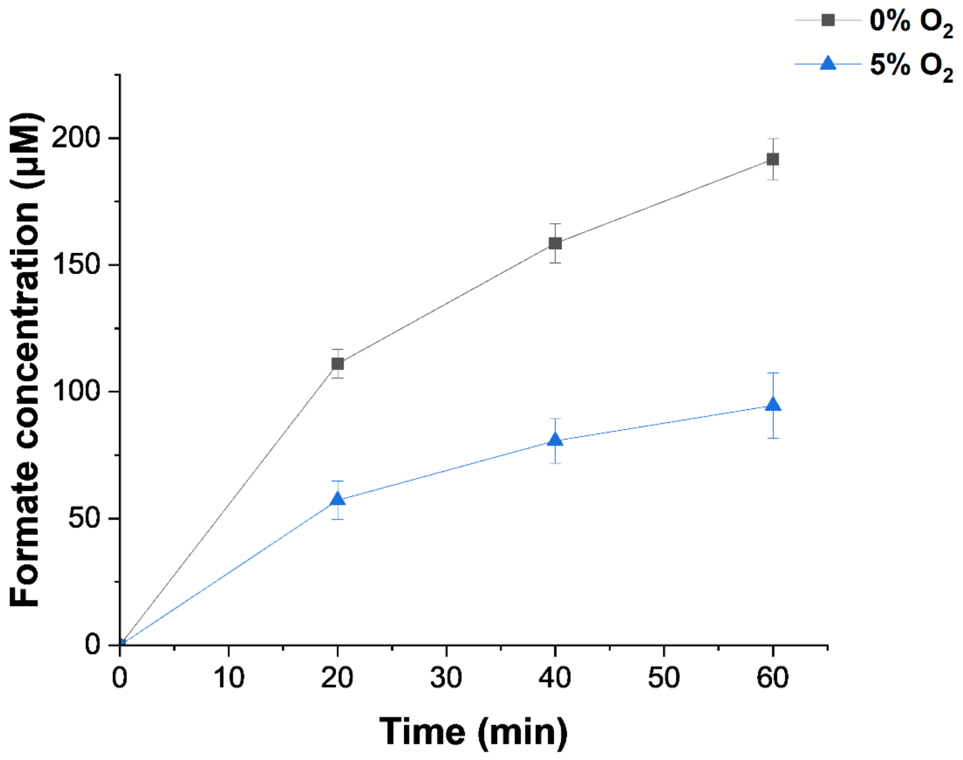

Supplementary Figure 4. H_2 and CO_2 conversion into formate by NAD^+-dependent ReSH and RcFDH cascade reactions under 0% and 5% O_2.
